# Development of a prognostic pyroptosis-related gene signature for head and neck squamous cell carcinoma patient

**DOI:** 10.1186/s12935-022-02476-3

**Published:** 2022-02-05

**Authors:** Weiwen Zhu, Jiayi Zhang, Mengyao Wang, Rundong Zhai, Yanbin Xu, Jie Wang, Mengqi Wang, Hang Zhang, Laikui Liu

**Affiliations:** 1 Department of Basic Science of Stomatology, The Affiliated Stomatological Hospital of Nanjing Medical University, 136# Hanzhong Road, Nanjing, 210029 Jiangsu China; 2 Jiangsu Province Key Laboratory of Oral Diseases, Nanjing Medical University, Nanjing, Jiangsu China; 3 Jiangsu Province Engineering Research Center of Stomatological Translational Medicine, Nanjing, Jiangsu China

**Keywords:** Head and neck squamous cell carcinoma, Pyroptosis, Prognostic model, NLRP3

## Abstract

**Objective:**

Head and neck squamous cell carcinoma (HNSCC) is a major threat to public health. Pyroptosis is a form of inflammatory programmed cell death that is still incompletely understood. The role of pyroptotic cell death in HNSCC remains to be fully defined. As such, the present study was developed to explore the potential prognostic utility of a pyroptosis-related gene (PRG) signature in HNSCC.

**Methods:**

PRG expression patterns and the associated mutational landscape in HNSCC were analyzed, after which a 6-gene prognostic model was constructed through least absolute shrinkage and selection operator (LASSO) and Cox regression analyses using the TCGA dataset, followed by validation with two GEO datasets (GSE41643 and GSE65858). The relative expression of the genes in the prognostic model was assessed via RT-qPCR in tumor and paired adjacent normal tissue samples from a 32-patient cohort. Potential predictors of patient outcomes associated with this 6-gene model were identified through topological degree analyses of a protein–protein interaction network. Moreover, the prognostic value of NLRP3 as a predictor of HNSCC patient prognosis was established through immunohistochemical (IHC) analyses of samples from 176 HNSCC patients. Lastly, in vitro studies were performed to further demonstrate the relevance of NLRP3 in the context of HNSCC development.

**Results:**

Differentially expressed PRGs were able to readily differentiate between HNSCC tumors and normal tissues. Risk scores derived from the 6-gene PRG model were independent predictors of HNSCC patient prognosis, and genes that were differentially expressed between low- and high-risk groups were associated with tumor immunity. RT-qPCR assays also showed the potential protective role of NLRP3 in HNSCC patients. IHC analyses further supported the value of NLRP3 as a predictor of HNSCC patient outcomes. Invasion and migration assays demonstrated the potential role of NLRP3 in the inhibition of HNSCC development.

**Conclusions:**

Overall, these results highlight a novel prognostic gene signature that offers value in the context of HNSCC patient evaluation, although additional research will be essential to elucidate the mechanisms linking these PRGs to HNSCC outcomes.

**Supplementary Information:**

The online version contains supplementary material available at 10.1186/s12935-022-02476-3.

## Introduction

Head and neck cancer is the sixth most prevalent form of cancer globally, with an estimated 830,000 diagnoses and 379,000 deaths occurring annually [1, 2]. Over 90% of these cases are diagnosed as head and neck squamous cell carcinoma (HNSCC), which arises from the mucosal epithelium of the oral cavity, pharynx, and larynx [3, 4]. Primary treatments for HNSCC patients include various combinations of surgery, radiotherapy, and chemotherapy depending on disease staging [5]. As there are no reliable approaches to systematically screening for this disease, a large percentage of HNSCC patients are diagnosed with advanced disease [6]. While there have been recent advances in treatment strategies for these patients, their 5-year survival rates remain low, underscoring the importance of developing new approaches to diagnosing and monitoring HNSCC in an individualized manner.

Biomarkers are specific indicators of particular physiological, pathological, or pharmacological processes that can enable effective patient identification and monitoring [7]. The advent of high-throughput sequencing technologies has spurred growing interest in the identification of specific biomarkers capable of guiding the prognostic evaluation and treatment of various cancers [8–10]. Many predictive biomarkers associated with HNSCC have been studied to date and may be of value as predictors of long-term patient prognosis and targeted treatment outcomes [11–13]. Most of these biomarkers, however, have yet to be subjected to any robust clinical validation or drug targeting efforts [14]. Prognostic biomarker models that are better able to predict HNSCC patient prognosis and treatment outcomes in a reliable manner are thus urgently needed to improve survival outcomes for affected individuals.

Pyroptosis is a form of inflammatory programmed cell death distinct from apoptosis [15]. Also known as cellular inflammatory necrosis, pyroptosis results in immunostimulatory molecule release from tumor cells [16]. At the molecular level, pyroptosis is associated with distinctive molecular features such as chromatin condensation, DNA fragmentation in the absence of DNA damage, and pore formation [17, 18]. Pyroptotic cell death has been suggested to play contrasting roles as an inhibitor or promoter of oncogenic progression, and it is closely correlated with cellular migration and proliferation [19–21]. Pyroptosis can be induced in response to specific microRNAs (miRNAs), chemotherapeutic drug treatments, or inflammasome activation, suppressing tumor growth [16]. Conversely, pyroptosis-related inflammatory cytokine production can give rise to a microenvironment that is better suited to tumor cell growth [22]. Pyroptosis-related gene (PRG) signatures can offer insight into prognostic outcomes across a range of cancer types [23, 24]. Treatment with TPL can activate GSDME-mediated pyroptosis by inhibiting the expression of HK-II in the mitochondria in HNSCC [25]. Although PRGs have been shown to have the prognostic value when predicting HNSCC patient outcomes [26], PRG-mediated immune infiltration and the aberrant changes in the biological behavior of cancer cells remain poorly understood.

Herein, we compared PRG expression between control and HNSCC tumor tissue samples to establish the value of these genes as predictors of patient outcomes. A novel PRG-based prognostic model for HNSCC was then developed and validated through both bioinformatics analyses of extant datasets and additional clinical samples.

## Materials and methods

### Data processing

RNA-sequencing (RNA-seq) data and matching clinicopathological findings pertaining to 506 patients with HSCC were downloaded from The Cancer Genome Atlas (TCGA) on Aug 10, 2021 (Additional file 1: Table S1). Somatic and copy number variation (CNV) data were downloaded from the TCGA and UCSC Xena websites. RNA-seq and clinical data used for external validation were downloaded from the GEO database (ID: GSE41643 and GSE65858). R (v 4.0.1) and the R Bioconductor packages were used for all analyses.

### Differentially expressed PRG identification

In total, the 33 PRGs listed in Additional file 2: Table S2 were identified based on prior reviews [16, 27]. Given that there were 31 annotated PRGs in the TCGA database, the differences in the expression of these genes between HNSCC patients and healthy controls were established using the ‘limma’ package. The Search Tool for the Retrieval of Interacting Genes (STRING) database was further utilized to construct a protein–protein interaction (PPI) network incorporating these PRGs based on a minimum interaction score of 0.7.

### Functional enrichment analyses

The R ‘ggplot2’ package was utilized to conduct Gene Ontology (GO) and Kyoto Encyclopedia of Genes and Genomes (KEGG) enrichment analyses.

### Prognostic PRG model development

A Cox regression analysis was used to assess the prognostic value of PRGs in HNSCC, with 6 of these significantly prognostic genes being retained for a subsequent prognostic model development through a LASSO Cox regression analysis. HNSCC patients from the TCGA cohort were separated into low- and high-risk groups based upon the median risk score value in this cohort, with Kaplan–Meier analyses subsequently being used to compare overall survival (OS) outcomes between these groups. Validation of this model was performed using the GSE41643 and GSE65858 datasets, and risk scores were calculated using the same formula employed for TCGA patient analyses.

### Real-time quantitative PCR (qPCR) analysis

RNA was extracted from cells using the Trizol reagent and reverse transcribed using the PrimeScript RT Reagent Kit (Takara Bio, Kusatsu, Japan). All qPCR analyses were performed in triplicate using the SYBR Green PCR Master Mix (Takara Bio) and detected using an Applied Biosystems 7900 Real-Time PCR System (Thermo Fisher Scientific, Waltham, MA, USA). The primer sequences used in this study are listed in Additional file 3: Supplementary Materials. Gene expression values were normalized to the endogenous control, and the 2^−ΔΔCt^ method was used for the relative quantification of gene expression. Primers used were as follows: GAPDH: forward 5′-GAAGGTGAAGGTCGGAGTC-3′ and reverse 5′-GAGATGGTGATGGGATTTC-3′; NLRP1: forward 5′-AGCTTCTGCTCGCCAATAAAG-3′ and reverse 5′-CCAGGTATGGAGGGCTAGGT-3′; NLRP2: forward 5′-TTCTGCGTCAAGCACTGTCG-3′ and reverse 5′-GGATCTCTCAACCTCGGCGT-3′; NOD2: forward 5′-CAATGACGATGCGGACACTG-3′ and reverse 5′-GCTGAATGGGAAGACAAAGAGAA-3′; PLCG1: forward 5′-CTCTATGGAATGGAATTTCGCC-3′ and reverse 5′-GGAGCCACCTCTCAATCTGC-3′; NLRP3: forward 5′-AGCACTAATCAGAATCTCACGCA-3′ and reverse 5′-TGTCTAATTCCAACACCTGAAGC-3′; IL-6: forward 5′-TGCCAGCCTGCTGACGAA-3′ and reverse 5′-AGCTGCGCAGAATGAGATGA-3′.

### Patients

In total, 176 primary HNSCC patients undergoing surgical treatment at the Department of Oral and Maxillofacial Surgery of the Affiliated Stomatological Hospital of Nanjing Medical University between 2010 and 2015 were recruited for this study. WHO classification criteria were used for tumor grading, while UICC and American Joint Commission on Cancer (AJCC) criteria were used for clinical and TNM grading. The Nanjing Medical University Ethics Committee approved this study, which was consistent with the Declaration of Helsinki. All patients provided written informed consent to participate.

### Immunohistochemical (IHC) staining

Paraffin-embedded HNSCC tumor tissue sections (4 μm) were treated with xylene for deparaffinization, rehydrated with an ethanol gradient, and treated for 20 min with 3% hydrogen peroxide. Following antigen retrieval, samples were blocked with normal goat serum and then probed overnight with anti- NLRP3 (Proteintech, 19771-1-AP). An HRP-polymer anti-rabbit Kit and a DAB Detection Kit (Fuzhou Maixin Biotech, Fuzhou, China) were then used to stain samples, and hematoxylin was employed for counterstaining. An ethanol gradient was then used to dehydrate samples, which were clarified with xylene and mounted using neutral gum.

### Pathological staining analyses

Two pathologists independently analyzed pathological samples. NLRP3 staining was assessed based upon immunoreactivity score (IRS) values calculated as: IRS = IS × PS. IS scoring was as follow: 0, negative; 1, weak; 2, moderate; 3, strong. PS scoring was as follows: 0, negative; 1, < 10%; 2, 11–50%; 3, 51–80%; 4, > 80% positive staining. Patients were then separated into low- and high-expression subgroups based on IRS scores of 0–4 and > 4, respectively.

### Cell proliferation assay

5-Ethynyl-2′-deoxyuridine (EdU) staining was used to assess proliferation. Briefly, cells were incubated with complete DMEM medium containing 50 μM EdU (RiboBio) at 37 °C in a 5% CO_2_ incubator for 2 h. Then, cells were washed with PBS, fixed with 4% paraformaldehyde for 30 min, neutralized with 50 μL 2 mg/mL glycine, and permeabilized using 0.5% Triton X-100. After washing with PBS, Apollo dye was added to each well, and then cells were incubated in the dark for 30 min at room temperature. Finally, Hoechst 33342 was applied for nuclear staining. Images were then captured using a fluorescence microscope (Leica Microsystems, Mannheim, Germany).

### Cell migration and invasion assays

Cell migration and invasion assays in vitro were performed using wound healing and transwell assays. In the wound healing assay, cells were plated in six-well plates and grown to 90% confluence. Artificial wounds were created in the monolayer surface using a 10 μl sterile pipette tip, and then the cells were then incubated in complete medium and allowed to migrate into the open wound area. Images of the same area of the wound were taken at 0, 12, and 24 h to determine the wound closure rate. Cell invasion assays were performed using Transwell inserts, and the Transwell inserts with the porous membrane (pore size 8 μm, Miliipore), which has been precoated with Matrigel (Corning, Bedford, MA, USA) for at least 1 h at 37 °C. Approximately 1 × 10^5^ HN6 cells or 2 × 10^5^ Cal27 cells were seeded per well in the upper chambers and incubated with complete medium in the lower chamber. After 24 h, the Transwell chambers were fixed with 4% PFA and stained with crystal violet (Sigma-Aldrich, St. Louis, MO, USA). Cells attached to the lower layer were then imaged (Olympus, Tokyo, Japan), every experiment was repeated for three times, and every chamber were counted in 5 randomly selected fields.

## Results

### Analysis of the PRG landscape in HNSCC

**Fig. 1:**
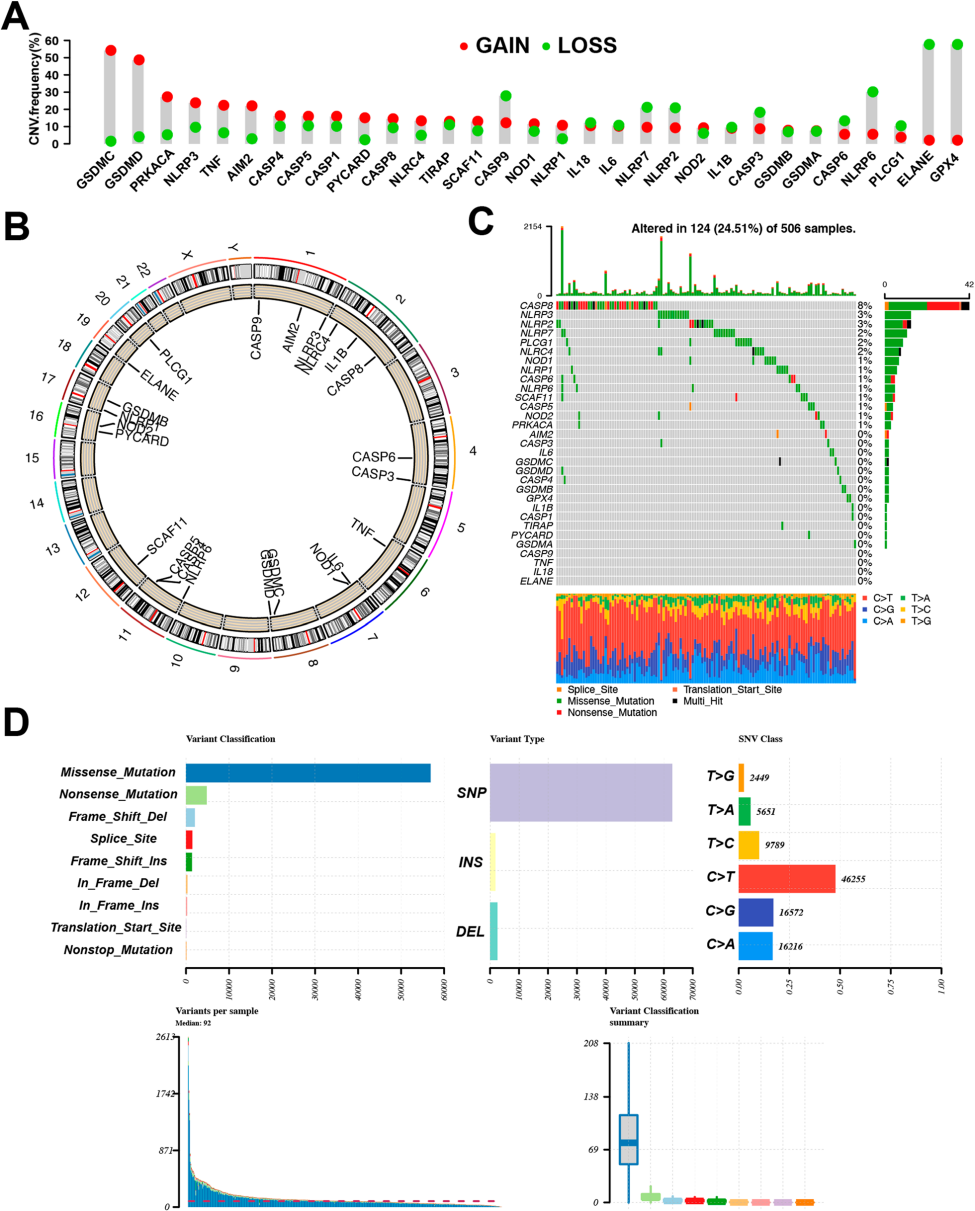
Landscape of the genetic and expression variation of PRGs in HNSCC. **A** The CNV frequency of 31 PRG in the HNSCC cohort. The height of the column represented the CNV frequency. **B** The location of PRGs on chromosomes in TCGA cohort. **C** The mutation frequency of 31 PRGs in HNSCC cohort. **D** The mutation classification of the 31 PRGs in TCGA cohort

**Fig. 2:**
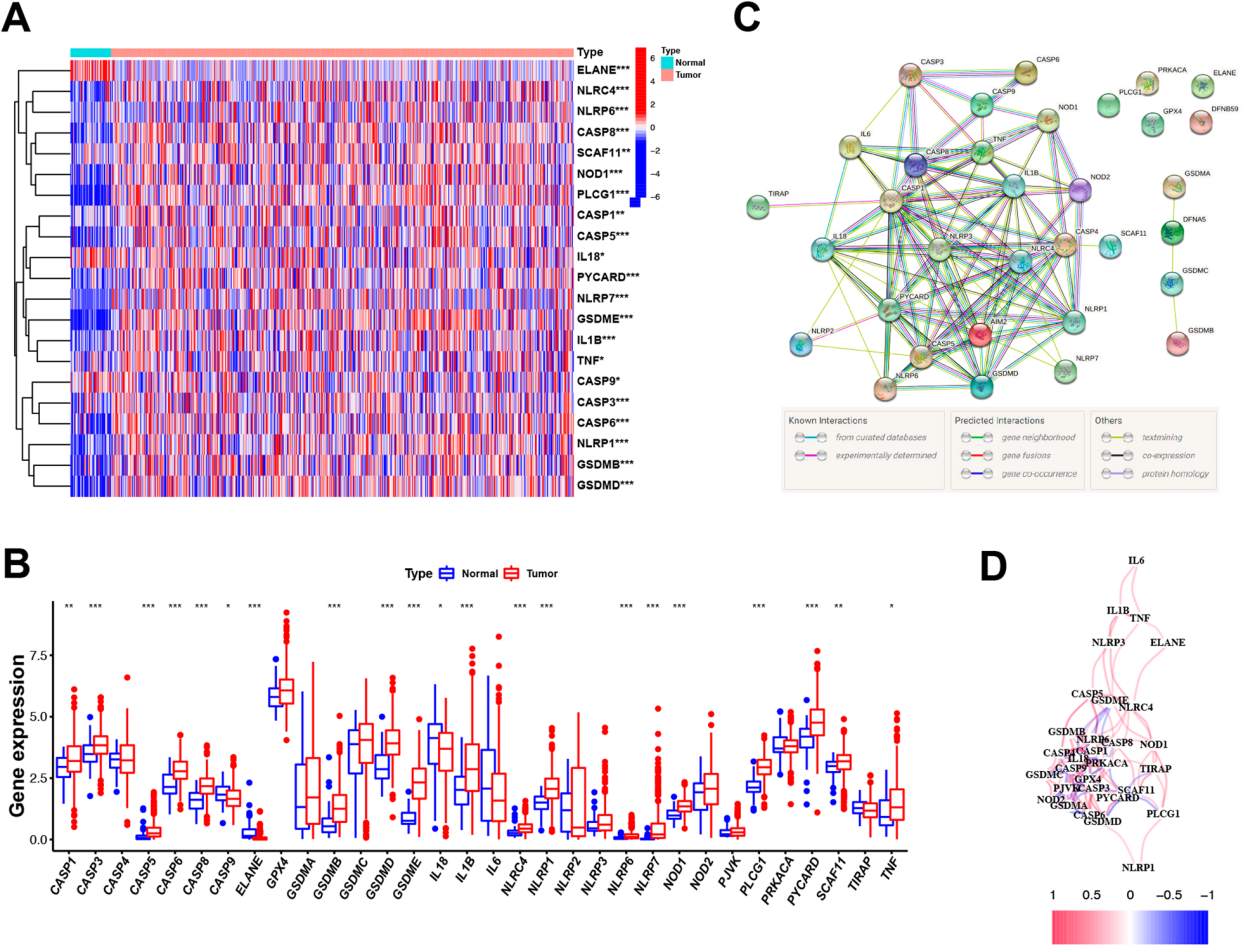
Expressions of the 31 pyroptosis-related genes and the interactions among them. **A** Heatmap (blue: low expression level; red: high expression level) of the pyroptosis-related genes between the normal (N, brilliant blue) and the tumor tissues (T, red). **B** The expression of 31 PRG in HNSCC and normal tissues, Tumor, red; Normal, blue. The upper and lower ends of the boxes represented the interquartile range of values. The lines in the boxes represented median value. **C** PPI network showing the interactions of the pyroptosis-related genes (interaction score = 0.7). **D** The correlation network of the pyroptosis-related genes (red line: positive correlation; blue line: negative correlation. The depth of the colors reflects the strength of the relevance). P values were showed as: *P < 0.05; **P < 0.01; ***P < 0.001

We began by assessing CNVs evident in 31 annotated PRGs from the TCGA HNSCC dataset, revealing alterations to be present in all of these genes. Of these, the highest CNV amplification frequency was observed for GSDMC, while the highest frequency of deletion was observed for GPX4 (Fig. 1A). The chromosomal locations of these PRGs were next established (Fig. 1B). Of 506 patients in the TCGA HNSCC cohort, 124 (24.51%) harbored somatic mutations in these PRGs. CASP8 was found to be mutated in 8% of these HNSCC patients, with missense and nonsense mutation being the most prevalent mutation type, thus suggesting a potential role of CASP8 in these HNSCC patients (Fig. 1C). Among all the mutations observed in identified PRGs, missense mutations were the most common (Fig. 1D), with C > T mutations being the most frequently detected form of single nucleotide polymorphism (SNP) in these patients. Next, PRG expression levels were compared between HNSCC tumors and control tissues, leading to the identification of 21 differentially expressed genes (DEGs) (Fig. 2A), of which 18 (CASP1, CASP3, CASP5, CASP6, CASP8, GSDMB, GSDMD, GSDME, IL-1B, NLRC4, NLRP1, NLRP6, NLRP7, NOD1, PLCG1, PYCARD, SCAF11, and TNF) were upregulated in tumors and three (CASP9, ELANE, and IL-18) were downregulated (Fig. 2B). A PPI network was then constructed to explore interactions among these PRGs in HNSCC (Fig. 2C), and a PRG correlation network was generated with positive and negative correlations being shown in red and blue, respectively (Fig. 2D). Overall, in light of CASP8 exhibiting the highest mutation rate among these PRGs and its observed upregulation in tumor tissues, the detailed etiological role of CASP8 in HNSCC development may warrant further study.

### PRG-based HNSCC patient clustering and functional enrichment analyses

**Fig. 3:**
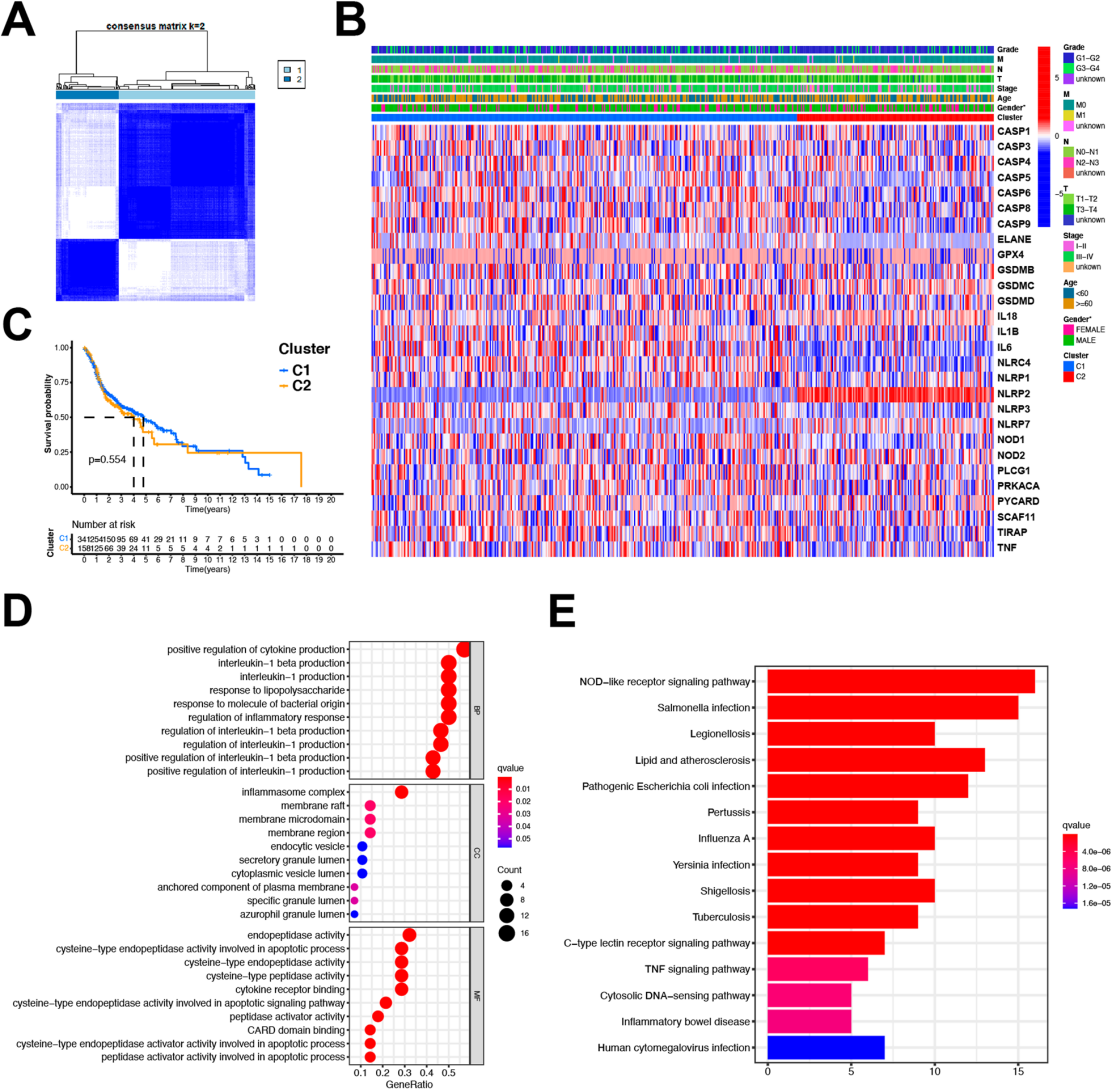
Tumor classifications and functional enrichments of PRGs in HNSCC. **A** 506 HNSCC patients were grouped into two clusters according to the consensus clustering matrix (k = 2). **B** Heatmap of the clinicopathologic characters of the two clusters classified by these DEGs. **C** Kaplan–Meier OS curves for the two clusters. **D** The enriched item in gene ontology analysis (BP biological process, CC cellular component, MF molecular function.). **E** The enriched item in Kyoto Encyclopedia of Genes and Genomes analysis. P value was showed as: *P < 0.05

To better understand the relationship between PRG expression and HNSCC, we conducted the consensus clustering of HNSCC patients from the TCGA dataset. Of tested values (2–10), we found that a clustering variable (k) value of 2 was associated with maximal intragroup correlations while maintaining low intergroup correlations. As such, these 506 HNSCC patients were effectively separated into two PRG-based clusters (Fig. 3A). When gene expression and clinical profiles were compared between these two clusters (C1 and C2), gender was the only discrepant feature (Fig. 3B). Overall survival (OS) did not differ significantly when comparing patients in these clusters (P = 0.554, Fig. 3C).

GO enrichment analyses of these 31 PRGs primarily revealed them to be associated with the positive regulation of cytokine production and interleukin-1 production (Fig. 3D). KEGG enrichment analyses further highlighted a role for these PRGs in the NOD-like receptor signaling and Salmonella infection pathways (Fig. 3E).

### PRG-associated prognostic model development

**Fig. 4:**
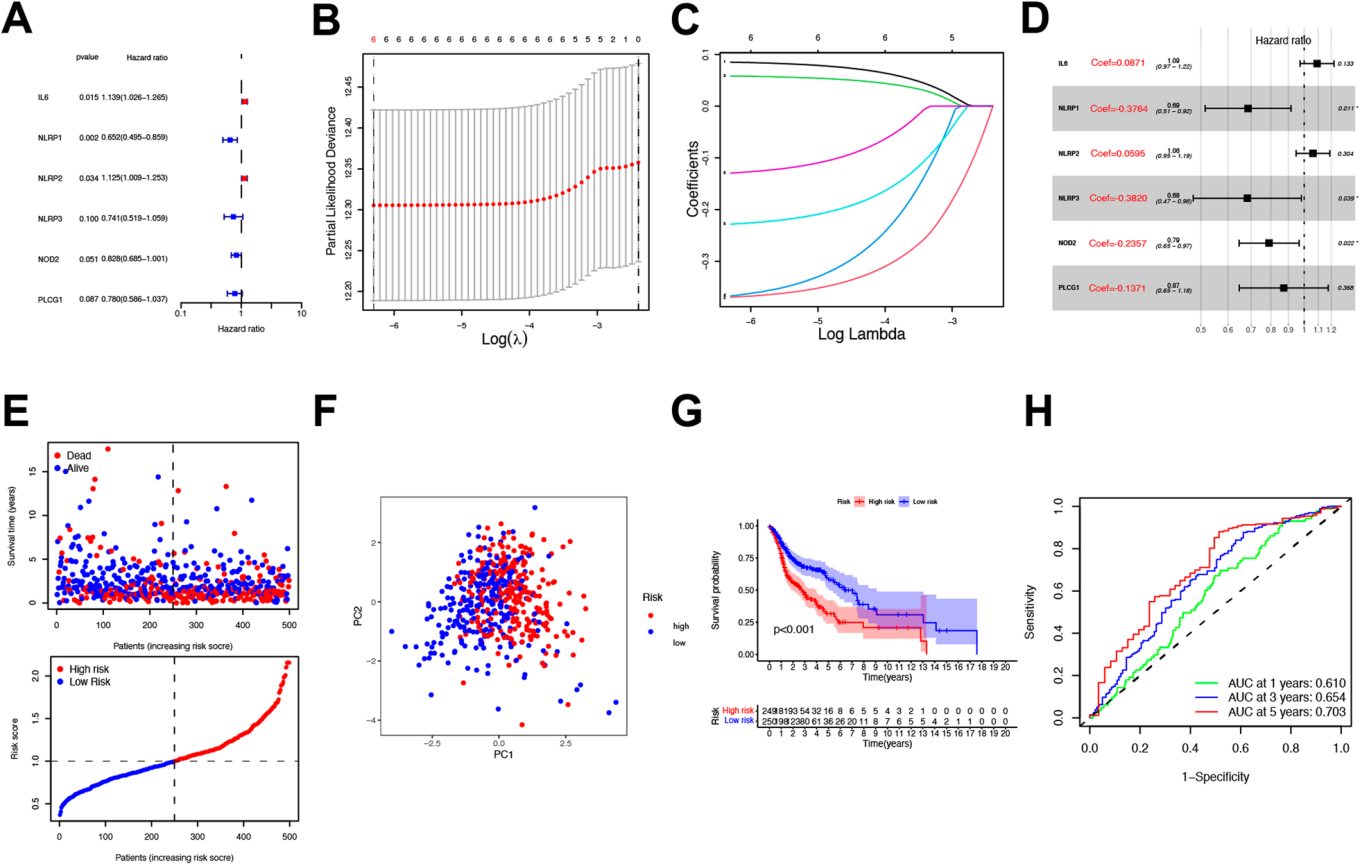
Construction of the risk signature in TCGA cohort. **A** Univariate cox regression analysis of OS for each pyroptosis-related gene, and 6 genes with P ≤ 0.1. **B** LASSO regression of the 6 OS-related genes. **C** Cross-validation for tuning the parameter selection in the LASSO regression. **D** Cox regression analysis for constructing the 6-gene prognostic model. **E** The survival status for each patient (low-risk population: on the left side of the dotted line; high-risk population: on the right side of the dotted line) (upper panel). Distribution of patients based on the risk score (lower panel). **F** PCA plot for HNSCC based on the risk score. **G** Kaplan–Meier curves for the OS of patients in the high- and low-risk groups. **H** ROC curves demonstrated the predictive efficiency of the risk score

Data pertaining to 500 HNSCC patients for whom survival outcomes were available in the TCGA cohort were used to guide prognostic model development. Initial univariate Cox regression analyses identified 6 PRGs (IL-6, NLRP1, NLRP2, NLRP3, NOD2, and PLCG1) that were significantly associated with patient survival (P ≤ 0.1), of which IL-6 and NLRP2 were associated with risk increases (HR > 1), whereas NLRP1, NLRP3, NOD2, and PLCG1 were protective (HR < 1) (Fig. 4A). Next, least absolute shrinkage and selection operator (LASSO) (Fig. 4B, C) and Cox regression analyses (Fig. 4D) were utilized to construct a 6-gene model based on an optimal λ value. This model was as follows: risk score = (0.0871*IL6 exp.) + (0.3764*NLRP1 exp.) + (0.0595*NLRP2 exp.) + (0.3820*NLRP3 exp.) + (0.2357*NOD2 exp.) + (0.1371*PLG1 exp.). These HNSCC patients were then stratified into low- and high-risk groups based upon median risk score values (Fig. 4E). In a principal component analysis (PCA), low- and high-risk patients were readily separated into two clusters (Fig. 4F). High-risk HNSCC patients exhibited poorer OS outcomes relative to low-risk patients (P < 0.001) (Fig. 4G). Time-dependent receiver operating characteristic (ROC) analyses were also utilized to assess the specificity and sensitivity of this model, revealing respective area under the ROC curve (AUC) values for 1-, 2-, and 3-year survival outcomes of 0.610, 0.654, and 0.703 (Fig. 4H). In summary, based on the Cox regression analysis, we further demonstrated the potential protective roles of NLRP1, NLRP3, and NOD2 as predictors of patient outcomes in a prognostic model.

### External risk signature validation

**Fig. 5:**
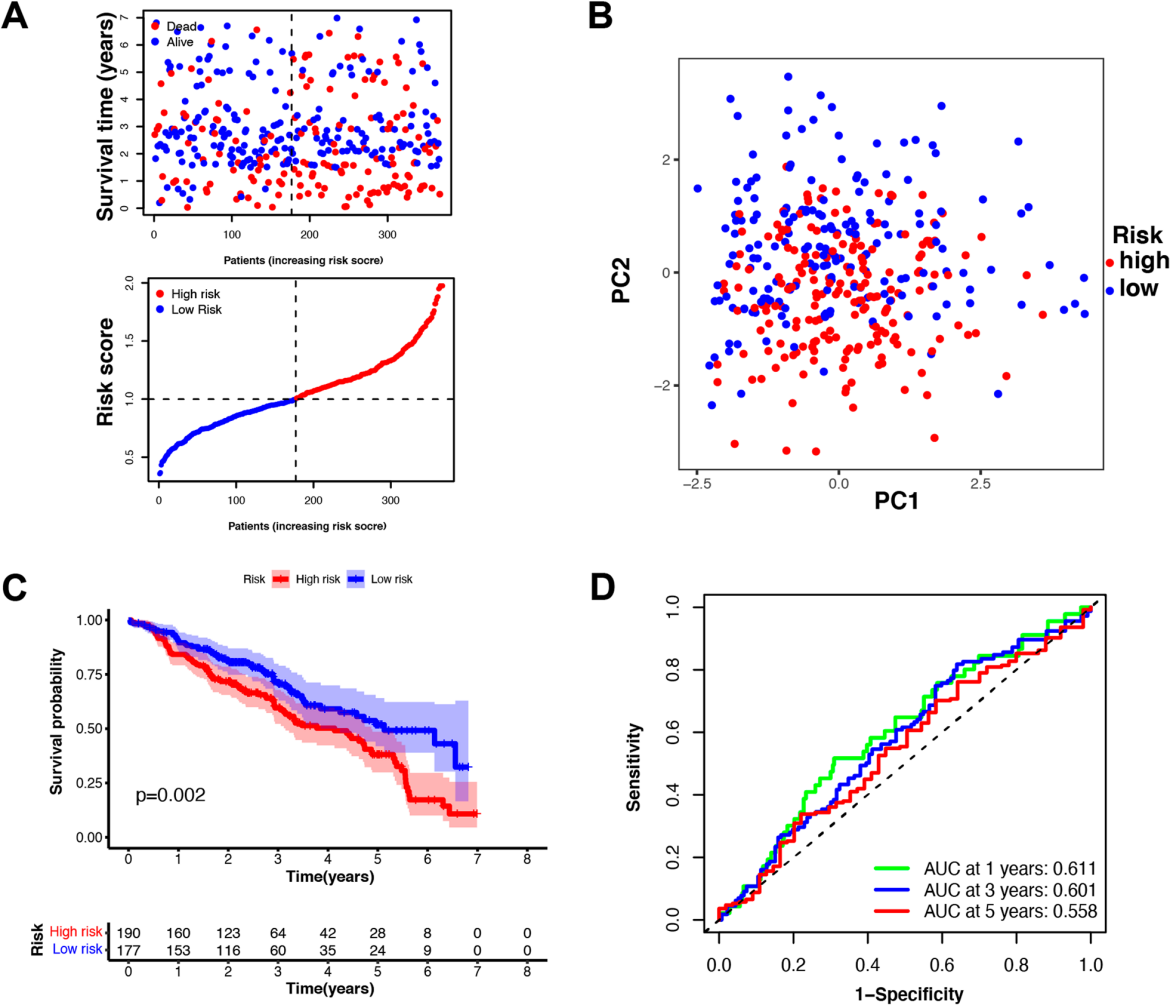
External validation of the risk signature in GEO cohort. **A** The survival status for each patient in validation cohort (low-risk population: on the left side of the dotted line; high-risk population: on the right side of the dotted line) (upper panel). Distribution of patients in the GEO cohort based on the median risk score in the TCGA cohort (lower panel). **B** PCA plot for HNSCC patients in GEO cohort. **C** Kaplan–Meier curves for comparison of the OS between low- and high-risk groups. **D** Time-dependent ROC curves for HNSCC

To validate the risk signature developed above, data pertaining to 367 HNSCC patients from the GSE41643 and GSE65858 datasets were utilized. Batch effects between these datasets were reduced with the ‘ComBat’ algorithm. These patients were then separated into high-risk (n = 190) and low-risk (n = 177) groups based upon the median risk scores from the TCGA cohort (Fig. 5A). PCA analysis revealed that these two subgroups were appropriately separated from one another (Fig. 5B). Significant differences in OS were evident between low- and high-risk HNSCC patient groups (P = 0.002) (Fig. 5C). ROC curve analyses further confirmed the predictive accuracy of this model, with respective AUC values pertaining to 1-, 2-, and 3-year survival outcomes of 0.611, 0.601, and 0.558 (Fig. 5D).

### Analysis of risk score prognostic value and predictive nomogram construction

**Fig. 6:**
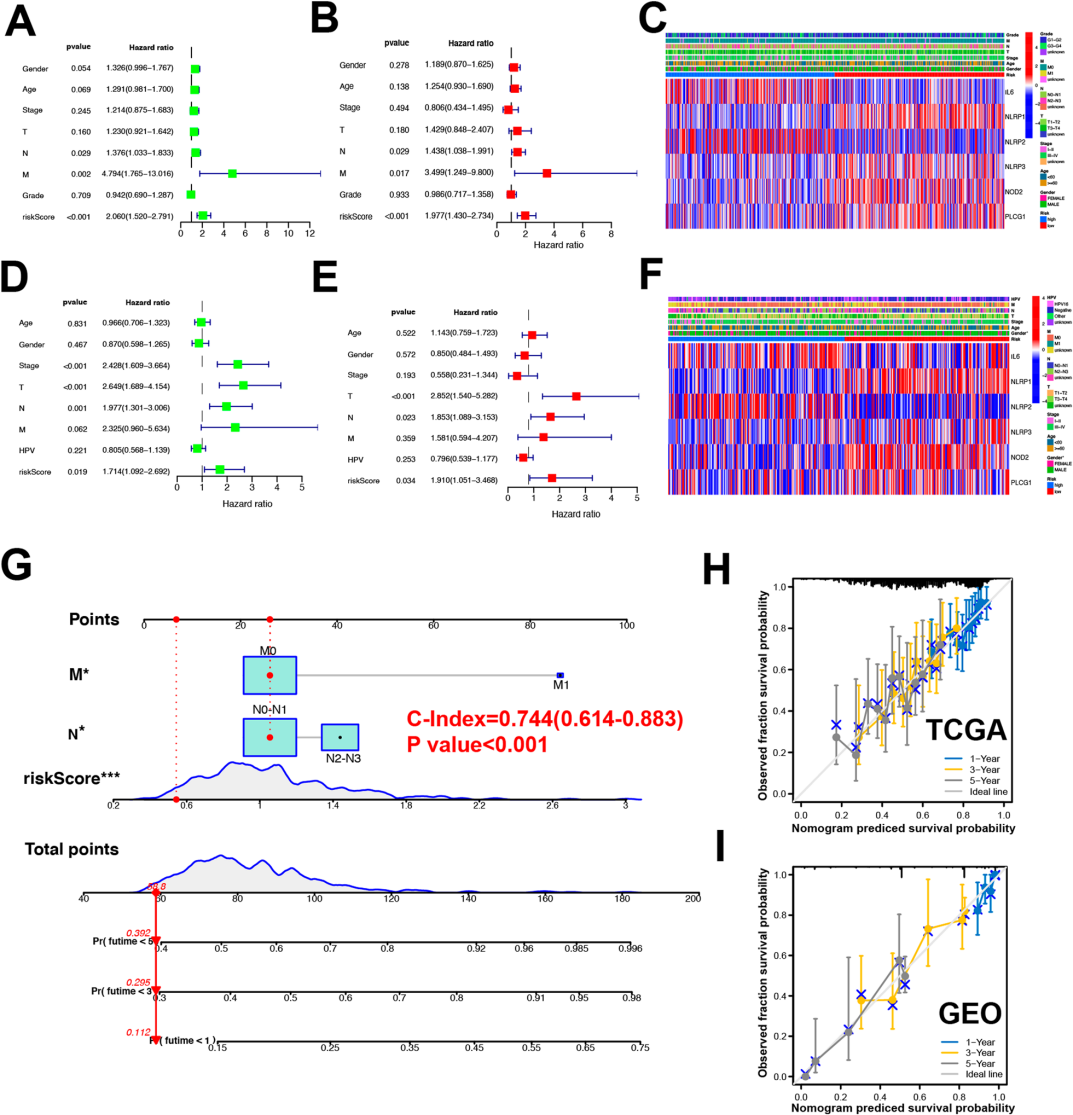
Construction of predictive nomogram and independent prognostic value of risk score. **A**, **B** Hazard ratio and P‐value of the constituents involved in univariate Cox regression analysis and multivariate analysis considering clinical the parameters and six prognostic PRGs in TCGA cohort. **C** Heatmap (blue: low expression; red: high expression) for the connections between clinicopathologic features and the risk groups in TCGA cohort. **D**, **E** Hazard ratio and P‐value of the constituents involved in univariate Cox regression analysis and multivariate analysis considering clinical the parameters and six prognostic PRGs in GEO cohort. **F** Heatmap for the connections between clinicopathologic features and the risk groups in GEO cohort.** G**–**I** Nomogram to predict the 1-year, 3-year, and 5-year overall survival rate of HNSCC patients. Calibration curve for the overall survival nomogram model in the discovery group. A dashed diagonal line represents the ideal nomogram. P values were showed as: *P < 0.05; ***P < 0.001

Univariate and multivariate analyses were next performed to explore the prognostic utility of the risk score developed above. In univariate analyses, this risk score was able to independently predict survival outcomes in the TCGA and GEO datasets (TCGA, HR = 2.060; GEO, HR = 1.714) (Fig. 6A, D). In multivariate analyses, risk scores were similarly able to independently predict HSCC patient prognosis (TCGA, HR = 1.977, GEO, HR: 1.910) (Fig. 6B, E). When a heatmap was constructed incorporating clinical findings from the TCGA (Fig. 6C) and GEO (Fig. 6F) cohorts, we observed diverse gender distributions between low- and high-risk patients in GEO cohorts (P < 0.05). We then constructed a nomogram incorporating all independent prognostic variables from multivariate regression analyses, and we found that this model was able to effectively predict 1-, 3-, and 5-tear OS in both GEO and TCGA cohorts (Fig. 6G–I). All these results further validated the accuracy of our model in the context of HNSCC patient prognostic evaluation.

### The relationship between prognostic PRGs and immune functionality

**Fig. 7:**
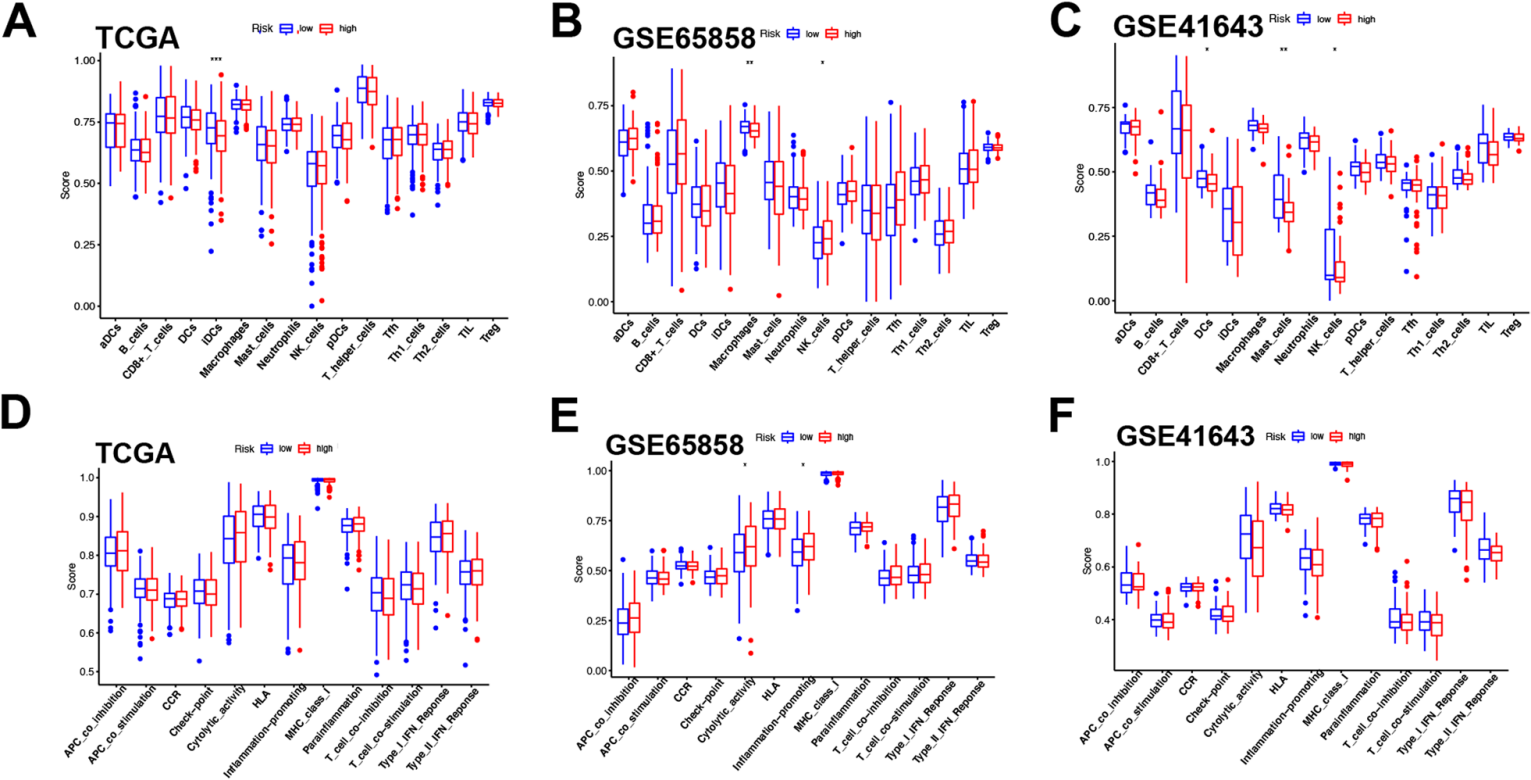
Comparison of the ssGSEA scores for immune cells and immune pathways. **A** Comparison of the enrichment scores of 16 types of immune cells between low- (blue box) and high-risk (red box) group in the TCGA cohort. **B**, **C** Comparison of the enrichment scores of the immune cells between low- and high-risk group in two GEO cohort. **D** Comparison of the enrichment scores of immune-related pathways between low- and high-risk group in the TCGA cohort. **E**, **F** Comparison of the enrichment scores of immune-related pathways between low- and high-risk group in the GEO cohort

Pyroptosis has been reported to be associated with the infiltration of the immune cells in various diseases. For example, PRG-induced macrophage infiltration was shown to be related to the release of inflammatory mediators, thereby inducing acute liver failure. Moreover, in the pyroptosis-activated immune microenvironment, CD8 + T cells and NK cells can both contribute to the generation of anti-tumor immunity [28, 29]. To better understand the crucial role of PRGs in the development of the tumor immune microenvironment, single-sample gene set enrichment analysis (ssGSEA) scores were next used to compare enrichment scores pertaining to 16 immune cell types and 13 immune-related pathways between high- and low-risk HNSCC patients in the TCGA and GEO validation cohorts used above. High-risk patients were found to exhibit decreased iDC infiltration in the TCGA cohort (Fig. 7A), while NK cell infiltration was increased and macrophage infiltration was decreased in high-risk patients from the GSE65858 cohort (Fig. 7B). No significant differences in immune status were evident in the TCGA or GSE41643 cohorts (Fig. 7D, F), while cytolytic activity and inflammation-promoting functionality were enriched among high-risk individuals in the GSE65858 cohort (Fig. 7E).

**Fig. 8:**
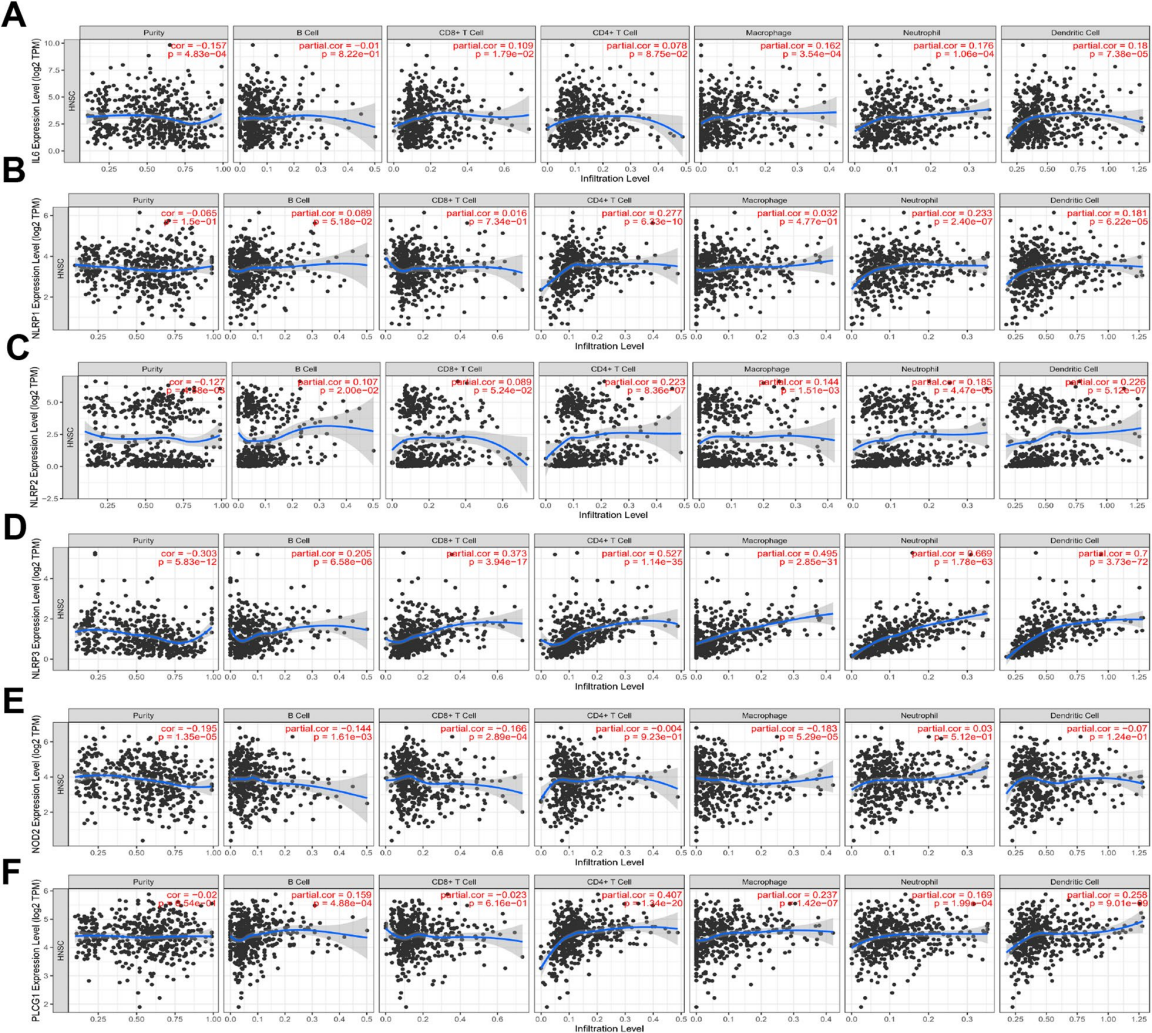
The association between six prognostic PRGs and immune infiltration (TIMER). The association between the abundance of immune cells and the expression of (**A**) IL-6, (**B**) NLRP1, (**C**) NLRP2, (**D**) NLRP3, (**E**) NOD2 and (**F**) PLCG1 in HNSCC

When correlations between the 6 prognostic PRGs defined above and immune infiltration were assessed with the TIMER database, NLRP3 was found positively correlated with B cell, CD8 + T cell, CD4 + T cell, macrophage, neutrophil, and dendritic cell abundance (Fig. 8).

### Assessment of differential PRG expression in HNSCC patients

**Fig. 9:**
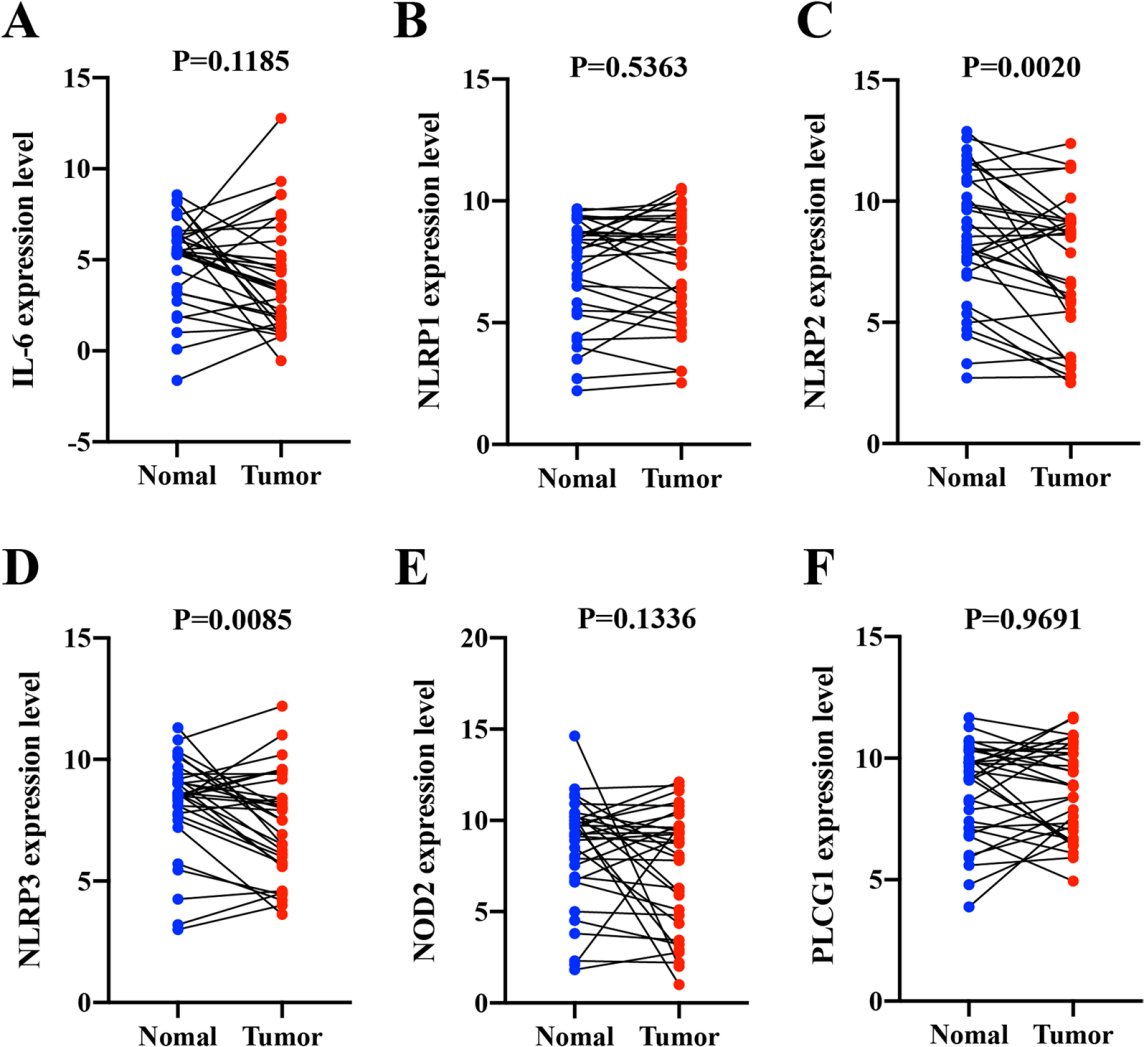
RT-qPCR analysis of six genes in prognostic model. Relative mRNA level of (**A**) *IL-6*, (**B**) *NLRP1* (**C**) *NLRP2* (**D**) *NLRP3* (**E**) *NOD2* and (**F**) *PLCG1* were assessed by RT-qPCR (Data was presented as mean ± SD)

Next, we recruited an independent cohort of 32 HNSCC patients consisting of 13 females and 19 males (40–66 years old), and sampled tumor tissues as well as paired normal epithelial tissues from these individuals for RT-qPCR analysis. After evaluating gene expression in our prognostic model, we demonstrated that *NLRP2* and *NLRP3* were significantly downregulated in HNSCC tissues in this patient cohort (Fig. 9). Given that *NLRP2* was significantly associated with increased risk level (HR > 1) and *NLRP3* was a protective gene (HR < 1) (Fig. 4A), our results further supported the independent prognostic value of NLRP3 when predicting HNSCC patient outcomes.

### Lower expression of NLRP3 is related to a poorer HNSCC patient prognosis

**Fig. 10:**
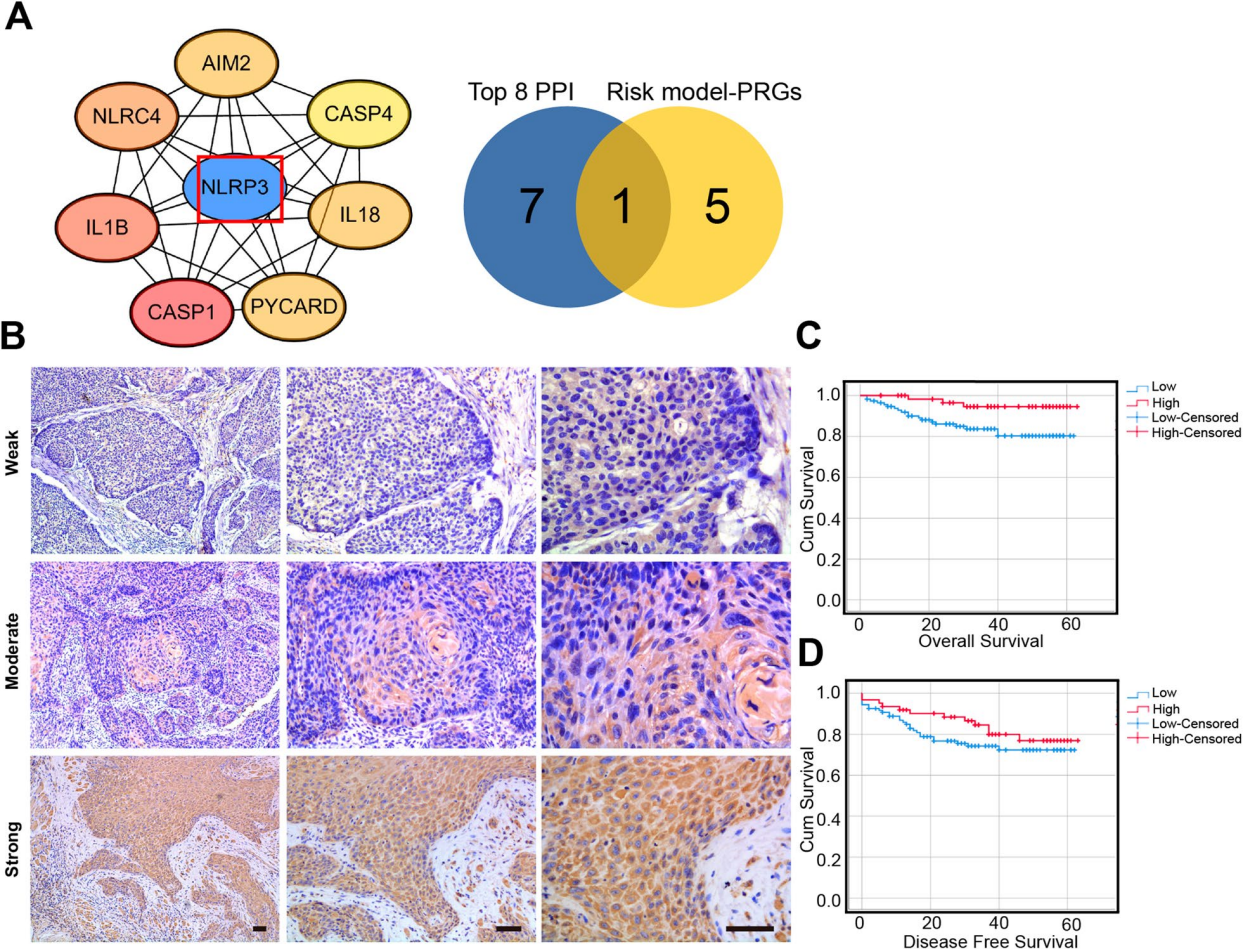
Lower expression of NLRP3 is associated with the poor prognosis of HNSCC patients. **A** The top 8 ranked proteins in topological degree in PPI network (Left panel), the venn diagram showed that NLRP3 was the only protein which existed in both PPI network and risk model (right panel). **B** NLRP3 expression in specimens of HNSCC patients. (n = 176). **C** OS curves for HNSCC patients with NLRP3-low and NLRP3-high expression (P = 0.019). **D** Kaplan–Meier analysis of DFS curves for HNSCC patients with NLRP3-low and NLRP3-high expression (P = 0.267)

Topological degree analyses of the 31 PRGs in the PPI network developed above led to the selection of the top 8 of these genes, of which only NLRP3 was included in our prognostic gene signature (Fig. 10A). Given that the hazard ratio of NLRP3 in the prognostic model was 0.68 (P = 0.039), we additionally conducted IHC analyses of NLRP3 staining in samples from 176 patients with HNSCC (Fig. 10B), the results demonstrated that the NLRP3 was significantly correlated with tumor size (P = 0.023). However, there was no significant relationship between NLRP3 expression and gender, age, tumor location, distant metastasis, pathological grade, infiltration, clinical stage, or recurrence (Additional file 4: Table S3). In addition, the lower expression level of NLRP3 was associated with the poor survival of HNSCC patients (Fig. 10C, D). Together, these findings confirm the prognostic utility of NLRP3 as a predictor of HNSCC patient survival.

### NLRP3 inhibits the invasion and migration of HNSCC cell lines

**Fig. 11:**
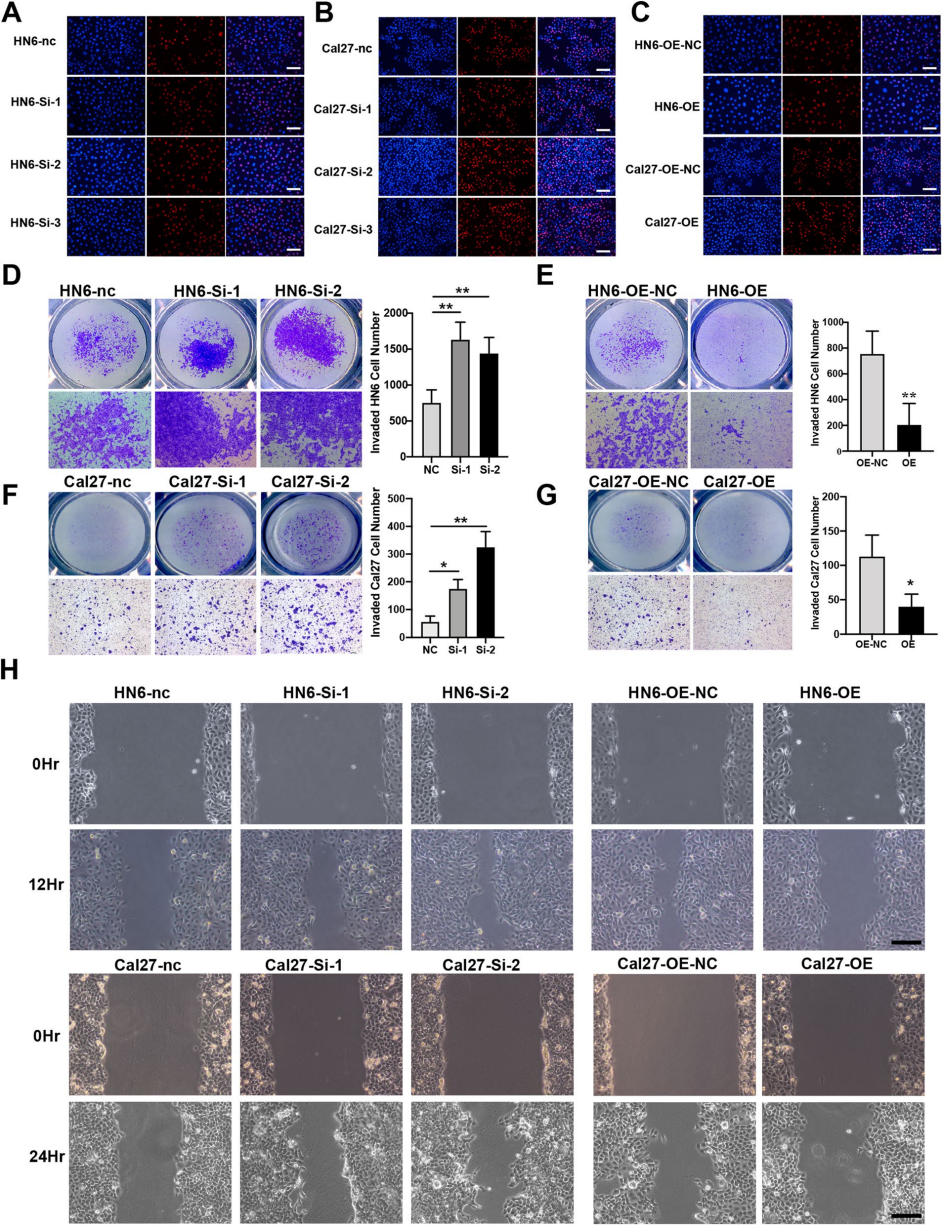
NLRP3 inhibits the invasion and migration of HNSCC cells. Small interferon RNAs (Si-RNAs) and pcDNA 3.1 plasmids were used to knockdown or overexpress (OE) the NLRP3 expression. **A**–**C** EdU assay was used to assess the proliferation of HNSCC cell lines. scale bar = 50 μm. **D**–**G** Transwell assays of HN6 and Cal27 cell lines were used to determine the invasion of HNSCC cells. **H** Wound healing assays of HN6 and Cal27 cell lines (OE, overexpression; Data was presented as mean ± SD, *P < 0.05, **P < 0.01) scale bar = 100 μm

Next, to demonstrate the effects of NLRP3 in the context of HNSCC development, we performed cell proliferation, invasion, and migration assays after knocking down or overexpressing NLRP3 in HN6 and Cal27 cell lines. These results indicated that the modulation of NLRP3 expression levels did not significantly affect cellular proliferation (Fig. 11A–C). However, Transwell assays demonstrated that NLRP3 knockdown resulted in enhanced cell invasion (Fig. 11D, F), whereas the overexpression of NLRP3 inhibited the invasion of HN6 and Cal27 cells (Fig. 11E, G). Wound healing assays also revealed that NLRP3 significantly inhibits cellular migration (Fig. 11H). Collectively, these results suggested that NLRP3 can act as a tumor suppressor via inhibiting the invasion and migration of HNSCC cells.

## Discussion

Normal physiological homeostasis is dependent on maintaining a balance between cellular proliferation, death, and differentiation [16]. While apoptosis and necrosis were once believed to be the primary forms of cell death, pyroptosis, autophagy, and other mechanisms have also been shown to mediate a loss of cellular viability [30]. Pyroptotic cell death is highly inflammatory and plays a role in a variety of pathogenic processes [31, 32]. Inflammasome activation initiates pyroptosis by recruiting caspase-1. Gasdermin D (GSDMD) serves as a caspase-1/11 substrate, inducing pyroptotic cell death owing to its ability to promote non-selective pore formation within the plasma membrane, subsequently driving cellular swelling, rupture, and the release of proinflammatory factors such as HMGB1, ATP, and IL-1β [16, 18, 33]. The relationship between pyroptosis and cancer is complex, given that this form of cell death can both drive tumor progression and impair antitumor immunity [18], while also inhibiting oncogenesis [34]. The mechanisms whereby PRGs affect HNSCC progression and survival outcomes remain to be clarified. Herein, we evaluated expression levels of 31 different PRGs in samples from HNSCC patients in the TCGA database, revealing 21 of these genes to be differentially expressed in HNSCC including 18 that were upregulated in this oncogenic setting. GO and KEGG analyses demonstrated a link between PRGs and cytokine production, IL-1 production, NLRP signaling, and Salmonella infection. These functional pathways were closely associated with HNSCC oncogenesis and progression. Salmonella infection, for example, can promote TLR4/ MyD88 pathway activation and consequent increases in macrophage and neutrophil infiltration [35]. In OSCC, targeting of the ROS/NLRP3 inflammasome/IL-1β signaling pathway has been suggested to improve outcomes associated with 5-FU adjuvant chemotherapy [36]. These data suggest that in-depth PRG studies may offer improved insight into antitumor immunity and inflammation.

HNSCC patients exhibit poor long-term survival outcomes, underscoring the need for more reliable biomarkers of long-term patient prognosis and treatment outcomes. TNM stage, vascular invasion, and other traditional clinicopathological biomarkers have yielded unsatisfactory outcomes when used to gauge patient prognosis [37]. RNA-seq and other high-throughput sequencing technologies have led to the identification of a wide variety of prognostic biomarkers associated with different cancers [38, 39]. Recent work has shown individual biomarkers are ill-suited to gauging cancer patient prognosis. A 4-gene immune-related biomarker signature (PVR, TNFRSF12A, IL21R, and SOCS1) may, together with other clinicopathological metrics, offer value as a means of assessing HNSCC patient prognosis [40]. Wang et al. assessed patterns of gene expression from 771 HNSCC patients in the TCGA and GEO databases, leading to the development of a 6-gene prognostic risk signature that was able to independently predict patient survival [41]. In a similar vein, we herein assessed the prognostic value of PRGs in HNSCC. We ultimately employed a LASSO Cox regression approach to construct a 6-PRG (IL-6, NLRP1, NLRP2, NLRP3, NOD2, and PLCG1) prognostic risk signature. Risk scores derived from this model were able to effectively stratify patients into low- and high-risk cohorts. High-risk patients exhibited worse survival outcomes than did low-risk patients. These findings thus confirm the prognostic value of this novel PRG risk signature, highlighting a novel approach to predicting HNSCC patient outcomes.

Cell death plays a central role in diverse pathological processes [42], with pyroptosis functioning as an inflammatory type of caspase-mediated cell death that can modulate the immunogenic potential of specific cancers [43]. Such immunogenicity is of critical importance in the context of tumor immunotherapy owing to the ability of tumor cells to activate a variety of immunosuppressive pathways within the local tumor microenvironment [44]. Pyroptosis can impact immune cell composition and associated immunological pathway activation to alter the processes governing tumorigenesis. Herein, we compared the immunological status of patients in our low- and high-risk groups, and assessed the relationship between the levels of difference prognostic PRGs in HNSCC (IL-6, NLRP1, NLRP2, NLRP3, NOD2, and PLCG1) and immune cell infiltration. The results of these analyses suggest that this prognostic gene signature may have important implications for immunotherapy treatment planning. NK cell infiltration was related to the activation of pyroptosis [45] and alternatively differentiated macrophage (M2 macrophage) infiltration was associated with the decrease of inflammation and pyroptosis [46]. Interestingly, In the present study, we noted that NK cell infiltration was increased and macrophage infiltration was decreased in high-risk patients from the GSE65858 cohort, these results are also in line with the previous studies [28, 29], indicating the distinct role of macrophages and NK cells in cancer development, and suggesting the potential role of immune cell-mediated activation of pyroptosis in affecting HNSCC patients outcome.

Among the 6 genes in our prognostic model, NLRP3 was positively correlated with immune infiltration in the TIMER database. The activation of NLRP3 has previously been reported in the context of metabolic changes, mitochondrial dysfunction, and the disassembly of the Golgi compartment. The NLRP3 inflammasome can also promote the caspase-1-dependent release of IL-1β and IL-18, which are inflammatory cytokines, in addition to activating downstream pyroptotic signaling mechanisms [47]. As the best-characterized inflammasome, NLRP3 is known to be associated with the infiltration of various types of the immune cells and the induction of immune cell-mediated tumor suppression in gastric cancer, hepatic cancer, and lymphoma [48–50]. Interestingly, our results are in line with those of previous studies, confirming the dual role of pyroptosis. Based on the Cox multivariate regression analysis of NLRP3 in our risk model, we posited that NLRP3 might act as a potential predictor of HNSCC patient outcomes (HR = 0.68, P = 0.039). In line with these reports, the results of our PPI network and survival analyses suggested that NLRP3 may be a valuable predictor of prognosis among HNSCC patients. Relative to another similar study [26], we developed a distinct PRG-based prognostic model and highlighted the potential role of NLRP3 as a predictor of HNSCC patient outcomes, while finding that lower levels of NLRP3 expression detected via IHC staining were associated with a worse prognosis. In vitro studies of invasion and migration activity also underscored the protective role of NLRP3 in the inhibition of HNSCC development. Overall, these data demonstrate the value of NLRP3 as a PRG that is independently associated with HNSCC patient survival and cancer development.

## Conclusions

In conclusion, the results of this study suggest that many differentially expressed PRGs were closely linked to the pathogenesis of HNSCC. Risk scores derived from our 6-gene PRG model were independent predictors of HNSCC patient outcomes, and genes that were differentially expressed between low- and high-risk groups were associated with tumor immunity. RT-qPCR analyses of tumor tissues also supported the protective role of NLRP3 in the context of HNSCC development, while IHC analyses further highlighted the value of NLRP3 as a predictor of HNSCC patient prognosis, and the expression of NLRP3 was additionally shown to affect the invasion and migration of HNSCC cells. Overall, these data highlight a novel gene signature that can be leveraged to predict survival outcomes in individuals with HNSCC, offering a foundation for future analyses of the link between PRGs and this cancer type.

## Supplementary Information

**Additional file 1:** Table S1.

**Additional file 2:** Table S2.

**Additional file 3:** Supplementary Materials.

**Additional file 4:** Table S3.

## Data Availability

The datasets used during the current study are available from the corresponding author on reasonable request.

## Abbreviations

HNSCC: Head and neck squamous cell carcinoma
PRGs: Pyroptosis-related genes
RNA-seq: RNA-sequencing
TCGA: The Cancer Genome Atlas
GEO: Gene expression omnibus
PPI: Protein–protein interaction
STRING: Search Tool for the Retrieval of Interacting Genes
GO: Gene ontology
BP: Biological process
CC: Cellular component
MF: Molecular function
KEGG: Kyoto Encyclopedia of Genes and Genomes
IHC: Immunohistochemistry
ssGSEA: Single-sample gene set enrichment analysis
RT-qPCR: Real-time-quantitative PCR
OE: Overexpression
EdU: 5-Ethynyl-20-deoxyuridine
