# Supplementary material for: Development of a prognostic pyroptosis-related gene signature for head and neck squamous cell carcinoma patient
Source: Cancer Cell Int. 2022 Feb 5;22:62. doi: 10.1186/s12935-022-02476-3 (PMC8817543; doi:10.1186/s12935-022-02476-3)
Supplement: Supplementary file 3 — Additional file 3: Supplementary Materials. [file 12935_2022_2476_MOESM3_ESM.docx]

**Supplementary Material**

**Development of a prognostic pyroptosis-related gene signature for head and neck squamous cell carcinoma patient**

**・Authors’ names:**

Weiwen Zhu^1,2,3,^^†^, Jiayi Zhang^1,2,3,†^, Mengyao Wang^1,2,3^, Rundong Zhai^1,2,3^, Yanbin Xu^1,2,3^, Jie Wang^1,2,3^, Mengqi Wang^1,^^2,3^, Hang Zhang^2,3^, Laikui Liu^1,2,3*^

**・Authors’ affiliations:**

^1^Department of Basic Science of Stomatology, The Affiliated Stomatological Hospital of Nanjing Medical University, Jiangsu, China;

^2^Jiangsu Province Key Laboratory of Oral Diseases, Nanjing Medical University, Jiangsu, China;

^3^Jiangsu Province Engineering Research Center of Stomatological Translational Medicine, Jiangsu, China.

^†^ Weiwen Zhu and Jiayi Zhang contributed equally to this work.

*** Corresponding author:**

Dr Lai-Kui Liu, Department of Basic Science of Stomatology, Affiliated Hospital of Stomatology, Nanjing Medical University. 136# Hanzhong Road, Nanjing, Jiangsu 210029, China. E-mail: [my_yunkong@njmu.edu.cn](mailto:my_yunkong@njmu.edu.cn)

**Materials and Methods**

**Western Blot**

Western blot was performed using methods previously described [1]. Briefly, cell lysates were separated on polyacrylamide–sodium dodecyl sulfate gel and electroblotted onto nitrocellulose membranes (Bio-Rad, Hercules, CA, USA). After blocking with 5% nonfat dry milk, the membranes were incubated with various antibodies overnight including NLRP3 (NBP2-12446, Novus) and Beta-Actin (66009-1-Ig, Proteintech). Then, membranes were incubated with a horseradish peroxidase–conjugated secondary antibody for 50 mins. The signals were visualized using ECL detection (Thermo Fisher Scientific).


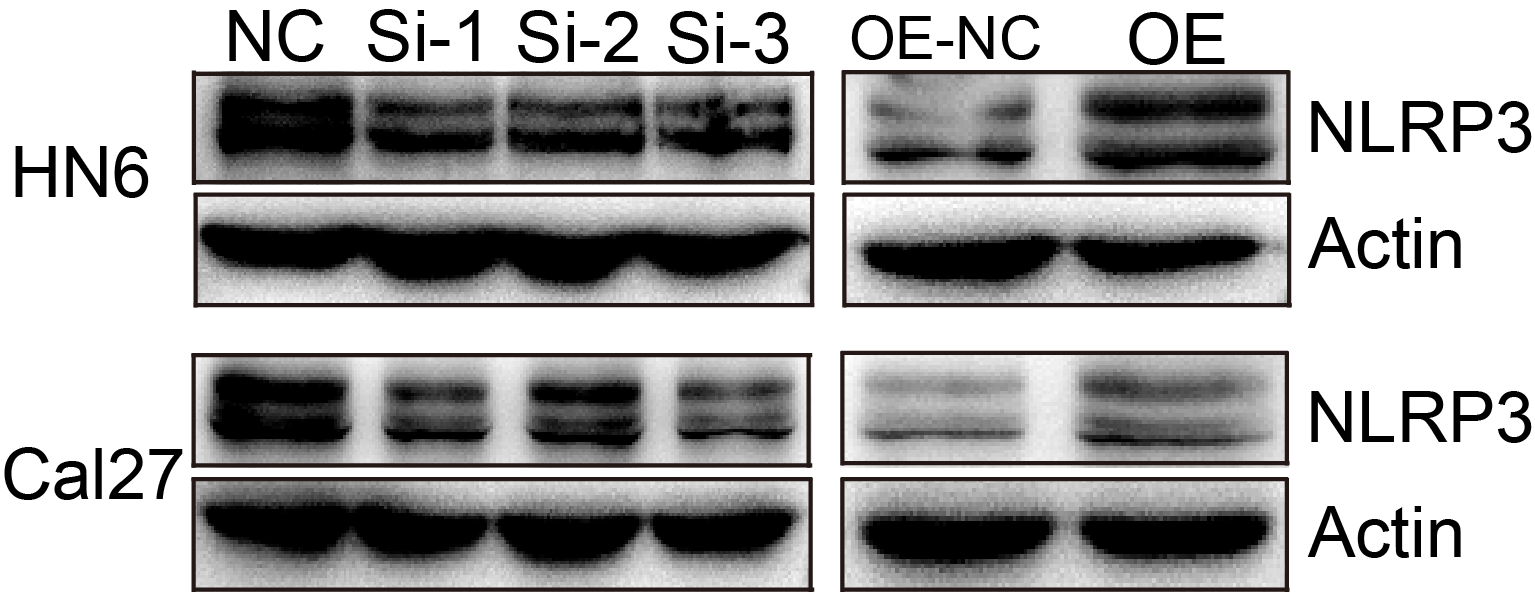


**Supplementary Figure S1. The knockdown and overexpression efficiency of NLRP3 in HN6 and Cal27 cells.** HN6 and Cal27 cells were transfected with small interferon RNA (siRNA) or pcDNA 3.1 for knockdown or overexpression of NLRP3. The protein expression level of NLRP3 was analyzed by western blot.

**Reference**

1. Zhu W, Xu R, Du J, Fu Y, Li S, Zhang P, Liu L, Jiang H: **Zoledronic acid promotes TLR-4-mediated M1 macrophage polarization in bisphosphonate-related osteonecrosis of the jaw**. *FASEB J* 2019, **33**(4):5208-5219.
