# Supplementary material for: Development of a prognostic pyroptosis-related gene signature for head and neck squamous cell carcinoma patient
Source: Cancer Cell Int. 2022 Feb 5;22:62. doi: 10.1186/s12935-022-02476-3 (PMC8817543; doi:10.1186/s12935-022-02476-3)
Supplement: Supplementary file 4 — Additional file 4: Table S3. [file 12935_2022_2476_MOESM4_ESM.docx]

|  |  | **NLRP3** |  |
| --- | --- | --- | --- |
| **Variable** | **Low** | **High** | **P Value** |
| **Age** |  |  |  |
| <50 | 20 | 10 | 0.812 |
| ≥50 | 94 | 52 |  |
| **Location** |  |  |  |
| Buccal | 30 | 21 | 0.282 |
| Central Carcinoma of the Jaw | 3 | 1 |  |
| Gingival | 15 | 15 |  |
| Lip | 6 | 1 |  |
| Oral Floor | 10 | 2 |  |
| Tongue | 39 | 16 |  |
| Palate | 6 | 2 |  |
| Other | 5 | 4 |  |
| **Gender** |  |  |  |
| Male | 69 | 39 | 0.757 |
| Femal | 45 | 23 |  |
| **Pathological Grade** |  |  |  |
| Ⅰ | 68 | 33 | 0.074 |
| Ⅱ | 28 | 25 |  |
| Ⅲ | 9 | 1 |  |
| Other | 9 | 3 |  |
| **Infiltration** |  |  |  |
| No | 41 | 26 | 0.436 |
| Yes | 73 | 36 |  |
| **T** |  |  |  |
| T1 | 18 | 10 | **0.023*** |
| T2 | 69 | 24 |  |
| T3 | 21 | 22 |  |
| T4 | 6 | 6 |  |
| **N** |  |  |  |
| N0 | 73 | 37 | 0.600 |
| N1 | 21 | 15 |  |
| N2 | 18 | 10 |  |
| N2b | 2 | 0 |  |
| **Clinical Stage** |  |  |  |
| Ⅰ | 14 | 10 | 0.113 |
| Ⅱ | 44 | 13 |  |
| Ⅲ | 33 | 25 |  |
| Ⅳ | 23 | 14 |  |
| **Recurrence** |  |  |  |
| No | 81 | 50 | 0.163 |
| Yes | 33 | 12 |  |
